# Predicting Disease Progression in Inoperable Localized NSCLC Patients Using ctDNA Machine Learning Model

**DOI:** 10.1002/cam4.70316

**Published:** 2024-10-24

**Authors:** Yuqi Wu, Canjun Li, Yin Yang, Tao Zhang, Jianyang Wang, Wanxiangfu Tang, Ningyou Li, Hua Bao, Xin Wang, Nan Bi

**Affiliations:** ^1^ Department of Radiation Oncology, National Cancer Center/National Clinical Research Center for Cancer/Cancer Hospital Chinese Academy of Medical Sciences and Peking Union Medical College Beijing China; ^2^ Geneseeq Research Institute Nanjing Geneseeq Technology Inc. Nanjing Jiangsu China; ^3^ State Key Laboratory of Molecular Oncology, National Cancer Center/National Clinical Research Center for Cancer/Cancer Hospital Chinese Academy of Medical Sciences and Peking Union Medical College Beijing China

**Keywords:** machine learning, MRD detection, neomer, non‐invasive, NSCLC

## Abstract

**Introduction:**

There is an urgent clinical need to accurately predict the risk for disease progression in post‐treatment NSCLC patients, yet current ctDNA mutation profiling approaches are limited by low sensitivity. We represent a non‐invasive liquid biopsy assay utilizing cfDNA neomer profiling for predicting disease progression in 44 inoperable localized NSCLC patients.

**Methods:**

A total of 97 plasma samples were collected at various time points during or post‐treatments (TP1: 39, TP2: 33, TP3: 25). cfDNA neomer profiling, generated based on target sequencing data, was used to fit survival support vector machine models for each time point. Leave‐one‐out cross‐validation (LOOCV) was performed to evaluate the models' predictive performances.

**Results:**

Our cfDNA neomer profiling assay showed excellent performance in detecting patients with a high risk for disease progression. At TP1, the high‐risk patients detected by our model showed an increased risk of 3.62 times (hazard ratio [HR] = 3.62, *p* = 0.0026) for disease progression, compared to 3.91 times (HR = 3.91, *p* = 0.0022) and 4.00 times (HR = 4.00, *p* = 0.019) for TP2 and TP3. These neomer profiling determined HRs were higher than the ctDNA mutation‐based results (HR = 2.08, *p* = 0.074; HR = 1.49, *p*&amp;#x02009;=&amp;#x02009;0.61) at TP1 and TP3. At TP1, the predictive model reached 40% sensitivity at 92.9% specificity, outperforming the mutation‐based method (40% sensitivity at 78.6% specificity), while the combination results reached a higher sensitivity (60%). Finally, the longitudinal analysis showed that the combination of neomer and ctDNA mutation‐based results could predict disease progression with an excellent sensitivity of 88.9% at 80% specificity.

**Conclusion:**

In conclusion, we developed a cfDNA neomer profiling assay for predicting disease progression in inoperable NSCLC patients. This assay showed increased predicting power during and post‐treatment compared to the ctDNA mutation‐based method, thus illustrating a great clinical potential to guide treatment decisions in inoperable NSCLC patients.

**Trial Registration:**

ClinicalTrials.gov: NCT04014465

## 1 Introduction

Lung cancer stands as the leading cause of cancer‐related deaths in China and the United States, with non‐small cell lung cancer (NSCLC) comprising ~89.4% and ~85.0% of these deaths, respectively [1, 2, 3, 4]. For NSCLC patients in the inoperable localized stage, the standard treatment protocol often involves a combination of chemotherapy and radiation therapy (CRT) [1, 2, 3]. However, despite receiving CRT, NSCLC prognosis remains unfavorable, with 5‐year survival rates distressingly low [1, 2, 3, 4]. Thus, the need arises for supplementary consolidation treatments, such as those identifying residual molecular disease (MRD) post‐treatment, to facilitate early prognosis estimation and the adjustment of treatment regimens according to disease progression.

Circulation tumor DNA (ctDNA), expelled into peripheral blood by tumor cells, is considered a non‐invasive biomarker. It can be detected and quantified to predict disease progression [5]. This notion is underscored by Yang et al., who found that the dynamic fluctuations in ctDNA—detected via targeted sequencing—were significantly associated with patients' disease progression following CRT treatments, with the predictive capacity peaking at 1 month [6]. However, the challenge of detecting MRD through ctDNA mutation profiling remains, given the low concentrations of ctDNA in patients' plasma samples post‐treatment. Though Wang et al. developed an ultrasensitive fragmentomics assay that utilizes whole‐genome sequencing (WGS)‐based cell‐free DNA (cfDNA) fragment profiling to detect MRD and predict recurrence more sensitively than traditional circulating mutations in post‐surgical NSCLC patients, there is room for improvement in the assay's sensitivity [7].

Georgakopoulos‐Soares et al. introduced the concept of neomers and defined it as short DNA sequences recurrent in tumor genomes yet absent in the reference human genome [8]. Thus, they are recognized as a type of cancer‐related biomarker. In their research, Georgakopoulos‐Soares et al. underscored the potency of cfDNA in locating neomers and diagnosing prostate cancer. Although neomer has previously been utilized in early detection research, it also shows promise for detecting MRD. This potential is supported by Wang et al., who demonstrated the use of other cfDNA biomarkers in landmark MRD detection [7].

In our study, we aim to evaluate the potential of non‐invasive liquid biopsy for the longitudinal monitoring of ctDNA in post‐CRT NSCLC patients and the estimation of their recurrence risk. We employed an ultra‐sensitive assay that leverages neomer features from serial plasma samples through targeted sequencing of 474 cancer‐related genes.

## 2 Materials and Methods

### 2.1 Patient Enrollment and Sample Collection

This study received approval from the Institutional Review Boards of Cancer Hospital, Chinese Academy of Medical Sciences (ethical number: 19/098‐1883). All patients provided written informed consent prior to sample collection. We enrolled 59 patients diagnosed with NSCLC at Cancer Hospital, Chinese Academy of Medical Sciences, in this prospective study between May 2018 and November 2020 (clinical trial number: NCT04014465).

The CRT regimen prescribed was a platinum‐based doublet, as recommended by the National Comprehensive Cancer Network (NCCN) guidelines. The regimen included combinations such as cisplatin plus etoposide, carboplatin plus paclitaxel, or cisplatin plus pemetrexed (exclusively for non‐squamous cell carcinomas).

We collected peripheral blood samples at various time points for analysis using targeted next‐generation sequencing (NGS) of 474 cancer‐related genes alongside whole genome sequencing. Specifically, after excluding patients who experienced disease progression (PD) events at the time of plasma sample collection and patients who received chemotherapy instead of CRT/radiotherapy (RT) due to liver/bone metastases or personal reasons, we had post‐treatment plasma samples from 44 out of the 59 patients. We evaluated the ctDNA status in plasma samples from these patients at three different time points prior to PD: during treatment (TP1: the fourth week of CRT/RT), post‐treatment landmark (TP2: 1 month after CRT/RT completion), and post‐treatment longitudinal (TP3: 3 months after CRT/RT completion). Specifically, 39 patients were assessed at TP1, 32 at TP2, and 25 at TP3. Among the 15 excluded patients, 4 lacked baseline samples, 8 did not receive CRT/RT, 2 only provided plasma samples at the time of progression, and 1 was lost to follow‐up. Among the 15 excluded patients, 4 did not have baseline samples, 8 did not receive CRT/RT, 2 only had plasma samples at PD, and 1 was lost during follow‐up.

In addition to ctDNA results, we collected detailed pathological and clinical response data for subsequent analysis. The median follow‐up time was 26.4 months.

### 2.2 Library Preparation and Sequencing

We utilized the DNeasy Blood & Tissue Kit (Qiagen) for plasma samples. The extracted DNA was quantified using the Qubit 3.0 fluorometer and dsDNA HS Assay Kit (ThermoFisher Scientific), following the manufacturer's instructions. For the plasma samples, we carried out a brief centrifugation to extract genomic DNA and remove cell debris. The supernatant was then used for the cfDNA extraction with the QIAamp Circulating Nucleic Acid Kit (Qiagen), per the manufacturer's instruction. Library preparations were conducted using the KAPA Hyper Prep Kit (KAPA Biosystems), according to the manufacturer's guidelines for different sample types.

In the case of targeted sequencing, we performed sequential operations of end‐repairing, A‐tailing, and indexed adapter ligation on 6.08–200 ng (median: 70.5 ng) of cfDNA or 1 μg of fragmented genomic DNA. This was followed by size selection using Agencourt AMPure XP beads (Beckman Coulter). The GeneseeqPrimeTM pan‐cancer gene panel (474 cancer‐relevant genes) was used for hybridization‐based target enrichment, covering ~1.5 Mb genomic regions (including ~0.93 Mb coding regions) using ~20,000 probes and xGen Lockdown Hybridization and Wash Reagents Kit (Integrated DNA Technologies). Captured libraries underwent on‐beads PCR amplification with Illumina p5 (50 AAT GAT ACG GCG ACC ACC GA 30) and p7 primers (50 CAA GCA GAA GAC GGC ATA CGA GAT 30) in KAPA HiFi HotStart ReadyMix (KAPA Biosystems), followed by quantification by qPCR using the KAPA Library Quantification Kit (KAPA Biosystems) and purification using Agencourt AMPure XP beads. Library fragment size was evaluated using the Bioanalyzer 2100 (Agilent Technologies). Sequencing of the target‐enriched library was subsequently conducted on the Illumina HiSeq4000 platform using PE150 sequencing chemistry (Illumina).

For WGS, we constructed libraries according to the manufacturer's protocol using the KAPA Hyper Prep Kit (KAPA Biosystems). Briefly, 5–10 ng of cfDNA per sample was subjected to end‐repairing, A‐tailing, and ligation with adapters sequentially. The Hamilton Microlab STAR automated liquid handling platform (Hamilton Company) was employed for the automated pipeline. The libraries were quantified with the KAPA SYBR FAST qPCR Master Mix (KAPA Biosystems) and underwent paired‐end sequencing on NovaSeq platforms (Illumina) per the manufacturer's guidelines.

### 2.3 Neomer Machine Learning Model

We processed the raw reads for the WGS data initially using Trimmomatic [9] for trimming, followed by PCR duplicate removal by the Picard toolkit (available at http://broadinstitute.github.io/picard/). We then mapped the reads onto the human reference genome (GRCh37/UCSC hg19) using the sequence aligner Burrows–Wheeler Aligner (BWA, v0.7.12) [10]. To minimize the potential impact of varying coverage among the WGS data on the model's predictive power, we down‐sampled the coverages to a unified 5X.

We employed neomer profiles as features to construct models, adapting the concept from previous reports. Georgakopoulos‐Soares et al. defined neomers as short DNA sequences that recur in tumor genomes but are absent from the human reference genome [8]. Upon surveying the PCAWG database (https://dcc.icgc.org/releases/PCAWG/), we identified a total of 977 recurrent single‐nucleotide polymorphisms (SNPs) from 2577 cancer patient samples. From these recurrent SNPs, we extracted 4616 neomers of 16 bp length. These neomers were then filtered against common population variants compiled in the Genome Aggregation Database (gnomAD v2) [11], resulting in a final total of 1758 neomers. For each plasma sample, we scanned the FASTQ data for exact matches to the 16 bp neomers of interest. The neomer features were profiled as the ratio of neomer‐detecting reads over the total reads and the read count of each of the 1758 neomers.

**FIGURE 1:**
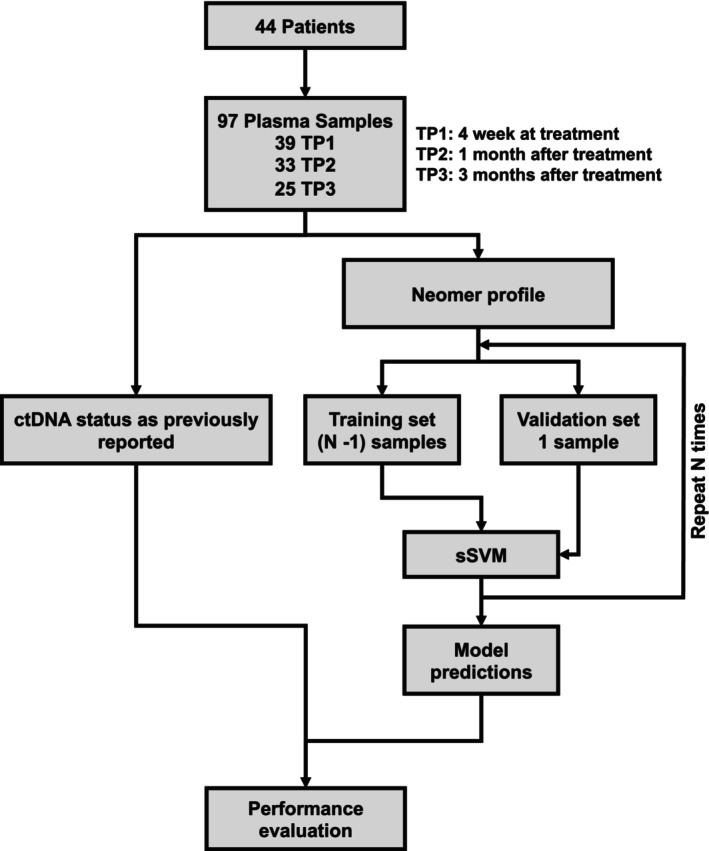
Flowchart of cohort and methodology. LOOCV, leave‐one‐out cross‐validation; sSVM, survival support vector machine; TP1, Timepoint 1, week 4 of CRT/RT; TP2, Timepoint 2, 1 month after CRT/RT; TP3, Timepoint 3, 3 months after CRT/RT.

A total of 97 plasma samples, including 39 TP1, 33 TP2, and 25 TP3, were collected from the 44 patients to develop MRD prediction models, as shown in Figure 1. For each time point, a machine learning model was constructed employing survival support vector machine (sSVM, scikit‐survival package, v0.17.2) algorithm [12] on the extracted neomer profiles. The sSVM algorithm was chosen based on many reasons: (1) It has shown high efficiency while dealing with high‐dimensional datasets; (2) The regularization parameter in the sSVM model is especially helpful in combating overfitting, which is a frequent issue in survival analysis due to limited sample size; (3) Researchers have shown that sSVM often outperforms traditional survival analysis models, such as the Cox proportional hazards model [12].

A leave‐one‐out cross‐validation (LOOCV) strategy was utilized by all three models for performance evaluation, following previous studies [7]. During the LOOCV procedure, a total of N (TP1:39, TP2: 33, TP3: 25) cross‐validation models were constructed using the entire dataset excluding the *i*th sample as the training cohort. Using the trained CV model, we predict the MRD risk for the *i*th sample that was left out. This process is repeated N times for every sample, ensuring each sample is tested exactly once. After obtaining the risk scores for all samples, their MRD status was then determined, by choosing a cutoff value to ensure 90% specificity.

### 2.4 Statistical Analysis

The sensitivity [true positive/(true positive + false negative)] and specificity [true negative/(true negative + false positive)], as well as the 95% confidence interval (exact binomial confidence limits), were calculated using the epiR package (v 2.0.19). The hazard ratio and the 95% confidence interval (Wald confidence interval) were calculated using the survival package (v 3.3.1). Cohen's Kappa and its 95% interval (bootstrapping method) were calculated using the psych package (v 2.3.6). All statistical analyses were performed in R (v.4.1.3).

## 3 Results

### 3.1 Study Design and Patient Characteristics

In our prospective study, we aimed to evaluate the clinical effectiveness of serial ctDNA monitoring in patients with NSCLC. Combined with the previous study, we initially enrolled 59 NSCLC patients in the discovery set. However, after excluding four patients who were unable to provide pre‐treatment plasma samples, eight patients who did not received CRT/RT, two patients who only had plasma samples at the time of progression, and one patient who was lost to follow‐up, our analysis focused on ctDNA data from the remaining 44 patients (Figure 1). This comprehensive baseline analysis provided an important foundation for our study.

We observed in the previous publication that the patient population was almost equally divided between those diagnosed with adenocarcinoma (ADC) at 45.5% and squamous carcinoma (SCC) at 50.9% [6] (data not shown). Further, it was notable that the majority of these patients (87.3%) had locally advanced disease, reflecting the severity of the conditions under investigation. We collected samples from 39 patients at the fourth week of CRT/RT (time point TP1), 33 patients at one‐month post‐CRT/RT (time point TP2), and 25 patients at 3 months post‐CRT/RT (time point TP3) (Figure 1).

### 3.2 Machine Learning Model for MRD Detection

Neomers are identified as short DNA sequences absent from the human genome but are commonly found in the genome of cancer patients [8, 13]. In addition, neomers exhibit a higher prevalence among individuals with cancer recurrence. In accordance with prior investigations, our findings indicate a significantly elevated ratio of mutated total reads in patients experiencing recurrence compared to those without recurrence in the TP1, TP2, and TP3 post‐CRT models (Figure S1). The mutated total reads ratio is the proportion of mutated reads relative to the total number of reads. Furthermore, as anticipated, there was a progressive elevation in cancer signals among patients with recurrence from TP1 to TP3, and patients experiencing relapse exhibited a statistically significant difference from patients who did not progress at TP3 (*p* = 0.025).

To predict the risk of recurrence, we developed machine learning models using the sSVM algorithm on the neomer feature profile at each of TP1, TP2, and TP3 post‐treatment. Each model employed LOOCV technique for evaluation and the selection of the cut‐off value was determined at 90% specificity of the training set.

As shown in Table 1, our neomer model showed consistent high performance across different time points. The post‐CRT model at TP1 demonstrated a sensitivity of 40% (95% CI: 21.1%–61.3%) and a specificity of 92.9% (95% CI: 66.1%–99.8%). At TP2, the model achieved a sensitivity of 40% (95% CI: 19.1%–63.9%) and a specificity of 92.3% (95% CI: 64%–99.8%). The model obtained a sensitivity of 45.5% (95% CI: 16.7%–76.6%) and a specificity of 92.9% (95% CI: 66.1%–99.8%) at TP3. Furthermore, as shown in Table S1, the sSVM algorithm outperformed other algorithms including Cox proportional hazards model (sensitivities: 12.0%–18.2%), Gradient Boosting Machine (sensitivities: 4.0%–18.2%) and Random Survival Forest (sensitivities: 0%–5.0%) at all three time points under the targeted 90% specificity cutoff.

**TABLE 1:** Performances of neomer model and mutation‐based ctDNA method.

TP1							TP2				TP3			
Neomer					Actual		Neomer		Actual		Neomer		Actual	
					Progress	Progress‐free			Progress	Progress‐free			Progress	Progress‐free
Predict			Progress		10	1	Predict	Progress	8	1	Predict	Progress	5	1
			Progress‐free		15	13		Progress‐free	12	12		Progress‐free	6	13
Sensitivity (95% CI)					40% (21.1%–61.3%)		Sensitivity (95% CI)		40% (19.1%–63.9%)		Sensitivity (95% CI)		45.5% (16.7%–76.6%)	
Specificity (95% CI)					92.9% (66.1%–99.8%)		Specificity (95% CI)		92.3% (64%–99.8%)		Specificity (95% CI)		92.9% (66.1%–99.8%)	
PPV (95% CI)					90.9% (58.7%–99.8%)		PPV (95% CI)		88.9% (51.8%–99.7%)		PPV (95% CI)		83.3% (35.9%–99.6%)	
NPV (95% CI)					46.4% (27.5%–66.1%)		NPV (95% CI)		50% (29.1%–70.9%)		NPV (95% CI)		68.4% (43.4%–87.4%)	
Accuracy (95% CI)					59.0% (42.1%–74.4%)		Accuracy (95% CI)		60.6% (42.1%–77.1%)		Accuracy (95% CI)		72.0% (50.6%–87.9%)	
Mutation					Actual		Mutation		Actual		Mutation		Actual	
					Progress	Progress‐free			Progress	Progress‐free			Progress	Progress‐free
Predict			Progress		10	3	Predict	Progress	9	0	Predict	Progress	2	2
			Progress‐free		15	11		Progress‐free	11	13		Progress‐free	9	12
Sensitivity (95% CI)					40% (21.1%–61.3%)		Sensitivity (95% CI)		45% (23.1%–68.5%)		Sensitivity (95% CI)		18.2% (2.28%–51.8%)	
Specificity (95% CI)					78.6% (49.2%–95.3%)		Specificity (95% CI)		100% (75.3%–100%)		Specificity (95% CI)		85.7% (57.2%–98.2%)	
PPV (95% CI)					76.9% (46.2%–95%)		PPV (95% CI)		100% (66.4%–100%)		PPV (95% CI)		50% (6.76%–93.2%)	
NPV (95% CI)					42.3% (23.4%–63.1%)		NPV (95% CI)		54.2% (32.8%–74.4%)		NPV (95% CI)		57.1% (34%–78.2%)	
Accuracy (95% CI)					53.8% (37.2%–69.9%)		Accuracy (95% CI)		66.7% (48.2%–82%)		Accuracy (95% CI)		56% (34.9%–75.6%)	
Neomer + Mutation		Actual		Neomer + Mutation		Actual		Neomer + Mutation				Actual		
		Progress	Progress‐free			Progress	Progress‐free					Progress	Progress‐free	
Predict	Progress	15	3	Predict	Progress	11	1	Predict			Progress	6	2	
	Progress‐free	10	11		Progress‐free	9	12				Progress‐free	5	12	
Sensitivity (95% CI)		60% (38.7%–78.9%)		Sensitivity (95% CI)		55% (31.5%–76.9%)		Sensitivity (95% CI)				54.5% (23.4%–83.3%)		
Specificity (95% CI)		78.6% (49.2%–95.3%)		Specificity (95% CI)		92.3% (64%–99.8%)		Specificity (95% CI)				85.7% (57.2%–98.2%)		
PPV (95% CI)		83.3% (58.6%–96.4%)		PPV (95% CI)		91.7% (61.5%–99.8%)		PPV (95% CI)				75% (34.9%–96.8%)		
NPV (95% CI)		52.4% (29.8%–74.3%)		NPV (95% CI)		57.1% (34%–78.2%)		NPV (95% CI)				70.6% (44%–89.7%)		
Accuracy (95% CI)		66.7% (49.8%–80.9%)		Accuracy (95% CI)		69.7% (51.3%–84.4%)		Accuracy (95% CI)				72% (50.6%–87.9%)		

Abbreviations: CI, confidence interval; NPV, negative predict value; PPV, positive predict value.

**FIGURE 2:**
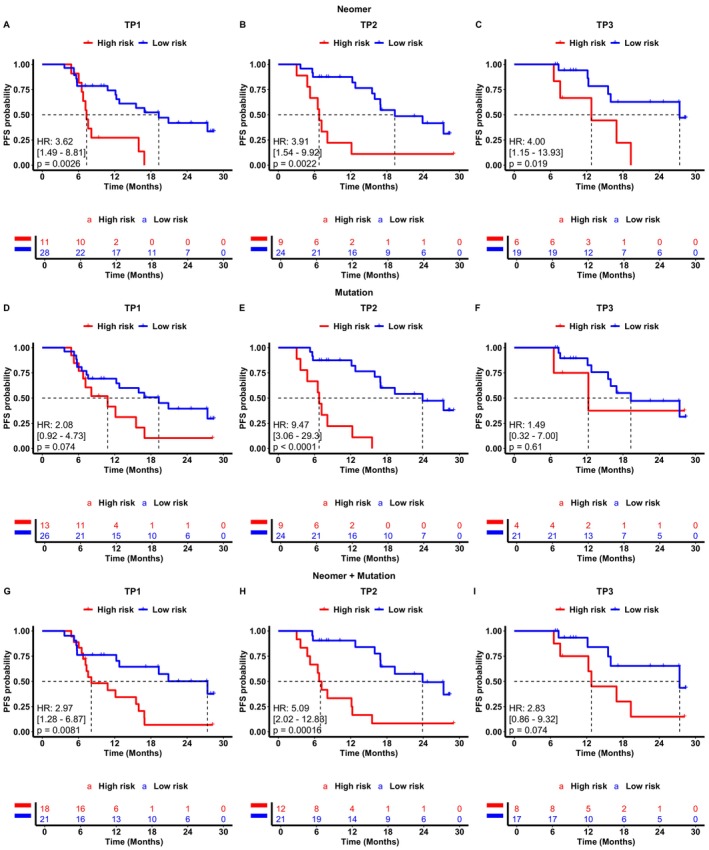
Progress‐free survival analysis in localized inoperable NSCLC patients using neomer and mutational methods. Kaplan–Meier curves of progression‐free survival stratified by the neomer model predicted status at (A) TP1, (B) TP2, (C) TP3. Kaplan–Meier curves of progression‐free survival stratified by ctDNA detection status at (D) TP1, (E) TP2, (F) TP3. Kaplan–Meier curves of progression‐free survival stratified by the combination of neomer model predicted and ctDNA detection status at (G) TP1, (H) TP2, (I) TP3. A combined high‐risk status was defined as being neomer model predicted high‐risk or ctDNA detection positive. TP1, Week 4 of CRT/RT; TP2, 1 month after CRT/RT; TP3, 3 months after CRT/RT.

**FIGURE 3:**
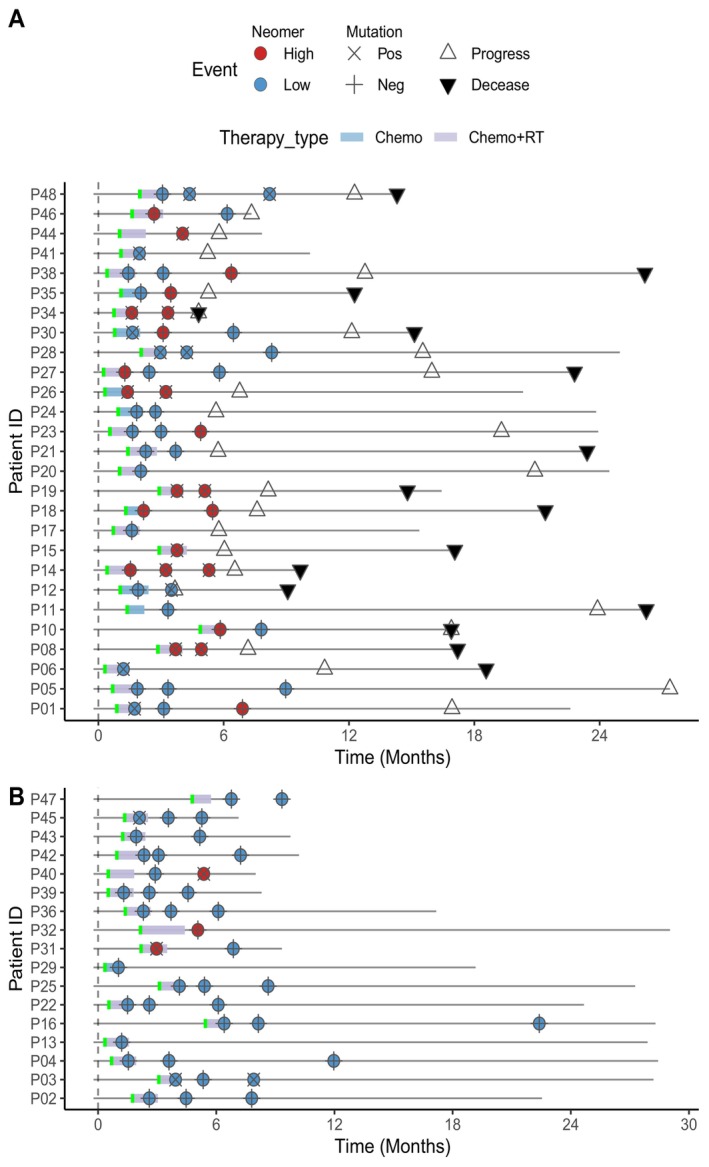
Neomer model prediction and ctDNA detection status prior to radiographic confirmed recurrence. Swim plots illustrating the neomer model predicted risk status, ctDNA mutation detection status and pathological events of cases in which relapse occurred (A) and not yet occurred (B) patients during the follow‐up.

Figure 2A demonstrates that our neomer model can provide stable predictions. At TP1, the neomer model displayed approximately 3.6 times higher risk than low‐risk patients (HR = 3.62, 95% CI: 1.49–8.81, *p* = 0.0026). At TP2, the model showed an approximately 3.9 times higher risk (HR = 3.91, 95% CI: 1.54–9.92, *p* < 0.0022; Figure 2B). Similarly, at TP3, the model predicted a 4 times higher risk (HR = 4.00, 95% CI: 1.15–13.93, *p* < 0.019; Figure 2C) in high‐risk patients. Furthermore, our model successfully identified 10 patients with recurrence as high risk at TP1 post‐treatment and predicted 2 patients with relapse as high risk at TP2 (Figure 3). Overall, the survival curves showed that our model could maintain its ability (mean HR = 3.84, standard deviation = 0.19) to distinguish high‐risk patients across different time points (Figure 2).

**FIGURE 4:**
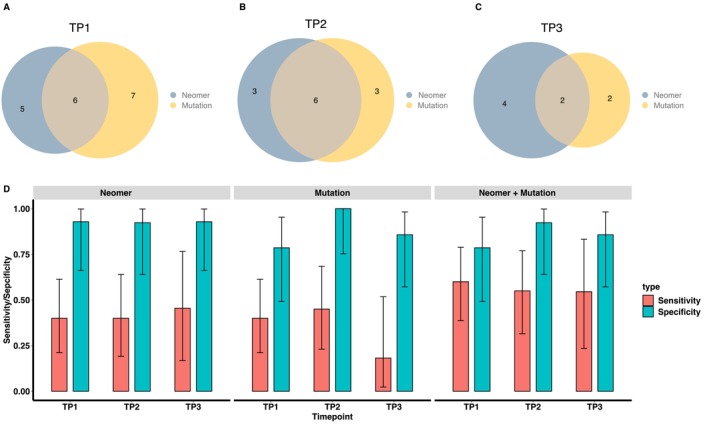
Enhanced sensitivities in detecting high‐risk patients for disease progression. Venn diagrams of neomer model prediction and ctDNA detection status for (A) TP1, (B) TP2, and (C) TP3. (D) Bar plots of sensitivities and specificities for detecting high progressive risk patients in the cohort. The error bars represent 95% confidence intervals. TP1, Week 4 of CRT/RT; TP2, 1 month after CRT/RT; TP3, 3 months after CRT/RT.

We then compared the outcomes predicted by our model with the results obtained from ctDNA mutation profiling. As shown in Table 1, the ctDNA mutation‐based method demonstrated a sensitivity of 40% (95% CI: 21.1%–61.3%) and a specificity of 78.6% (95% CI: 49.2%–95%) at TP1, a sensitivity of 45% (95% CI: 23.1%–68.5%) and a specificity of 100% (95% CI: 75.3%–100%) at TP2, and a sensitivity of 18.2% (95% CI: 2.28%–51.8%) and a specificity of 85.7% (95% CI: 57.2%–98.2%) at TP3 post‐treatment. Figure 4 demonstrated a significant overlap between the model and the ctDNA mutation‐based method, indicating mutual predictions of high‐risk patients. At TP3, the neomer model identified 6 high‐risk patients, and mutation profiling detected 4 ctDNA‐positive patients, with 2 patients predicted as high‐risk by both methods. This resulted in a sensitivity 2.5 times higher for the neomer model (Table 1). Furthermore, the neomer model showed fair agreement with the ctDNA mutation‐based methods at all three time points, illustrating Cohen's Kappas of 0.2800 (95% CI: 0–0.5979), 0.5416 (95% CI: 0.2189–0.8645) and 0.2574 (95% CI: 0–0.6941) for TP1, TP2 and TP3, respectively.

Patients with positive ctDNA status had an increased risk of approximately 2.1 (HR = 2.08, 95% CI: 0.92–4.73, *p* = 0.074; Figure 2D) at TP1, 9.5 times (HR = 9.47, 95% CI: 3.06–29.3, *p* < 0.0001; Figure 2E) at TP2 and 1.5 times (HR = 1.49, 95% CI: 0.32–7.00, *p* = 0.61; Figure 2F) at TP3, respectively, which showed more fluctuation compared to the high‐risk patients predicted by the neomer model. The performances of ctDNA mutation profiling of all patients and patients presented in three time points were inferior to the performances of neomer models (Figure S2C). At the condition of all patients, the neomer model predicted 19 patients with a high risk of recurrence, compared to the 18 ctDNA‐positive patients by mutation profiling, as shown in Figure S2A. A different trend was observed in patients present in three time points, with the neomer model predicting 6 patients with a high risk of recurrence compared to the 7 ctDNA positive patients by mutation profiling, yielding very similar ratios of sensitivity (Figure S2A,B). Figure S3 demonstrated that the factor of neomer + mutation in TP1 (*p* = 0.007; Figure S3A) and in TP2 (*p* = 0.004; Figure S3B) was found to be the most significant factor affecting recurrence risk. However, it is not significant in TP 3(0.117; Figure S3C). Notably, age also plays an essential role during the TP2 (*p* = 0.013), significantly influencing the recurrence risk (Figure S3B). Likewise, in the condition of all patients (Figure S4A) and patients present in three time points (Figure S4B), the factor of neomer + mutation is an essential factor impacting the recurrence risk, with *p* = 0.003 and *p* = 0.013, respectively.

A patient was labeled as high risk for progression if they were classified as high risk by either the model or had a positive ctDNA status based on target panel sequencing. For a low progression risk status, a patient needed to have an expected low‐risk status according to the neomer model and a negative ctDNA status according to mutational profiling. Combining the two methods increased sensitivities at all three time points while maintaining similar specificities (Figure 3D). As shown in Table 1, the combined‐method model achieved a sensitivity of 60% (95% CI: 38.7%–78.9%), 55% (95% CI: 31.5%–76.9%), and 54.5% (95% CI: 23.4%–83.3%) with a specificity of 78.6% (95% CI: 49.2%–95.3%), 92.3% (95% CI: 64%–99.8%), and 85.7% (95% CI: 57.2%–98.2%) at TP1, TP2, and TP3, respectively. As shown in Table 1, the combined method showed higher overall accuracies at three time points (TP1, TP2 and TP3) with trend toward significance than both the neomer model (*t*‐test, *p* = 0.0931) and the ctDNA mutation‐based method (*t*‐test, *p* = 0.0564).

When aggregating the results from TP1, TP2, and TP3 and considering all the patients at these time points, the combined model reached a sensitivity of 77.8% (95% CI: 57.7%–91.4%, Table 1) at a specificity of 70.6% (95% CI: 44%–89.7%) and patients labeled high risk had an increased risk of 4.51 compared to low‐risk patients (HR = 4.51, 95% CI: 1.77–11.51, *p* = 0.0007; Figure S5E).

When considering only the 19 patients present at all three time points, a combination of the neomer and mutation‐based models resulted in an even higher sensitivity of 88.9% (95% CI: 51.8%–99.7%, Table S2) at a specificity of 80% (95% CI: 44.4%–97.5%), with 13.37 times higher risk in high‐risk patients (HR = 13.37, 95% CI: 1.63–109.4, *p* = 0.0022; Figure S5F). In comparison to a single mutation‐based method, which yielded hazardous ratios (HR) ranging from 3.25 to 3.99, our neomer model exhibited HR ranging from 3.12 to 9.11. Notably, the integration of these two methods resulted in an HR of 13.37 across 19 patients assessed at three different time points. This indicates the potential predictive efficacy of the integrated model when longitudinal patient data is available for analysis at multiple time points.

Furthermore, we performed a bootstrapping analysis (*n* = 1000) using a total of 57 samples from the 19 patients who were assessed at all three time points. As shown in Figure S6, the overall accuracies generated from the longitudinal data were significantly higher (Wilcoxon test, *p* < 2.2e^−16^) than the overall accuracies by the independent time points during the bootstrapping analysis.

## 4 Discussion

Our study indicates that using non‐invasive liquid biopsies for the longitudinal monitoring of ctDNA could serve as a reliable prognostic marker for post‐CRT NSCLC patients. Notably, our cohort consisted of inoperable localized NSCLC patients, a subgroup that often faces limited treatment options and poor prognoses. By focusing on these patients, our research addresses a critical gap in monitoring and managing their disease. Our utilization of an ultra‐sensitive assay that leverages neomer features in serial plasma samples showed promising results in estimating recurrence risk in this cohort. Our tumor‐agnostic assay offers an option for patients with inoperable localized NSCLC by eliminating the requirement for prior tumor tissue samples, unlike the current tumor‐informed methods.

Our approach is underpinned by the use of ctDNA, a non‐invasive biomarker that can be detected and quantified to predict disease progression. The potential of ctDNA as a prognostic marker is well‐established, with studies such as that by Yang et al., indicating a significant association between dynamic fluctuations in ctDNA and disease progression following CRT treatments [6]. However, the sensitivity of conventional mutation tracking technique for monitoring MRD using ctDNA is constrained by several factors, including tumor burden and the fraction of ctDNA [14, 15, 16, 17, 18]. Here, we built upon this foundational research by utilizing neomers, a novel concept introduced by Georgakopoulos‐Soares et al., which are short sequences recurrent in tumor genomes but identified as neomers in the human genome [13]. We showed that the detection of neomers in the ctDNA potentially indicates disease recurrence in NSCLC patients. This demonstrated the potential of neomer as a biomarker for ctDNA monitoring and recurrence risk estimation in post‐CRT NSCLC patients and the application of neomer could significantly enhance our ability to adapt treatment regimens according to disease progression. We further revealed that the combination of neomer and machine learning models outperforms mutations alone. Finally, compared to fragmentomics biomarkers that require WGS, neomers exhibit great advantages as an additional biomarker for the existing targeted sequencing data.

However, our study has several limitations. Firstly, it should be noted that our study is based on existing data, and thus a prospective study is essential to assess the real‐world applicability of neomer in MRD. Secondly, the small sample size hinders our ability to include an independent validation set, necessitating the use of LOOCV to validate the performance of our machine learning model. This approach, along with k‐fold cross‐validation and repeated k‐fold cross‐validation, has been shown to provide validation power and mitigate overfitting. Lastly, the utilization of targeted sequencing may underestimate the performance of the model, as it only includes cancer‐related genes in the panel, whereas the use of WGS could potentially enhance the performance.

To overcome the limitations of a small sample size and the lack of an independent validation cohort in the current study, we have initiated a prospective study aimed at substantiating the predictive power of our model in real‐world settings. It is essential to increase the sample size through robust recruitment strategies, multi‐center collaborations, and partnerships with healthcare providers. Importantly, the inclusion of a separate validation cohort from diverse geographical regions will enhance the robustness and applicability of our predictive model. Incorporating these approaches will address the current limitations and pave the way for more comprehensive and applicable research outcomes.

In conclusion, our study successfully evaluated the potential of non‐invasive liquid biopsy as a longitudinal monitoring tool for circulating tumor DNA (ctDNA) in post‐CRT NSCLC patients. This approach enabled us to estimate the recurrence risk of these patients. Furthermore, our findings contribute to the growing evidence supporting the utility of neomer in assessing ctDNA dynamics and providing valuable prognostic information in the context of post‐CRT NSCLC management.

## Author Contributions

**Yuqi Wu:** conceptualization (equal), data curation (equal), formal analysis (equal), supervision (equal), writing – review and editing (equal). **Canjun Li:** formal analysis (equal), writing – original draft (equal). **Yin Yang:** formal analysis (equal), writing – original draft (equal). **Tao Zhang:** data curation (equal), investigation (equal). **Jianyang Wang:** data curation (equal), investigation (equal). **Wanxiangfu Tang:** software (equal), visualization (equal). **Ningyou Li:** writing – original draft (equal). **Hua Bao:** software (equal), visualization (equal). **Xin Wang:** supervision (equal), writing – review and editing (equal). **Nan Bi:** conceptualization (equal), project administration (equal), supervision (equal), writing – review and editing (equal).

## Ethics Statement

This study received approval from the Institutional Review Boards of Cancer Hospital, Chinese Academy of Medical Sciences (ethical number: 19/098‐1883).

## Consent

All patients provided written informed consent prior to sample collection. All authors read and approved the final version of the manuscript.

## Conflicts of Interest

Wanxiangfu Tang, Ningyou Li and Hua Bao are employees of Nanjing Geneseeq Technology Inc., Nanjing, Jiangsu, China. The remaining authors declare no conflicts of interest.

## Supporting information


Figure S1.

Table S1.

Table S2.

## Data Availability

The datasets used and analyzed during the current study are available from the corresponding author upon reasonable request.
