# Supplementary material for: Predicting Disease Progression in Inoperable Localized NSCLC Patients Using ctDNA Machine Learning Model
Source: Cancer Med. 2024 Oct 24;13(20):e70316. doi: 10.1002/cam4.70316 (PMC11499892; doi:10.1002/cam4.70316)
Supplement: Supplementary file 1 — Figure S1. [file CAM4-13-e70316-s001.docx]

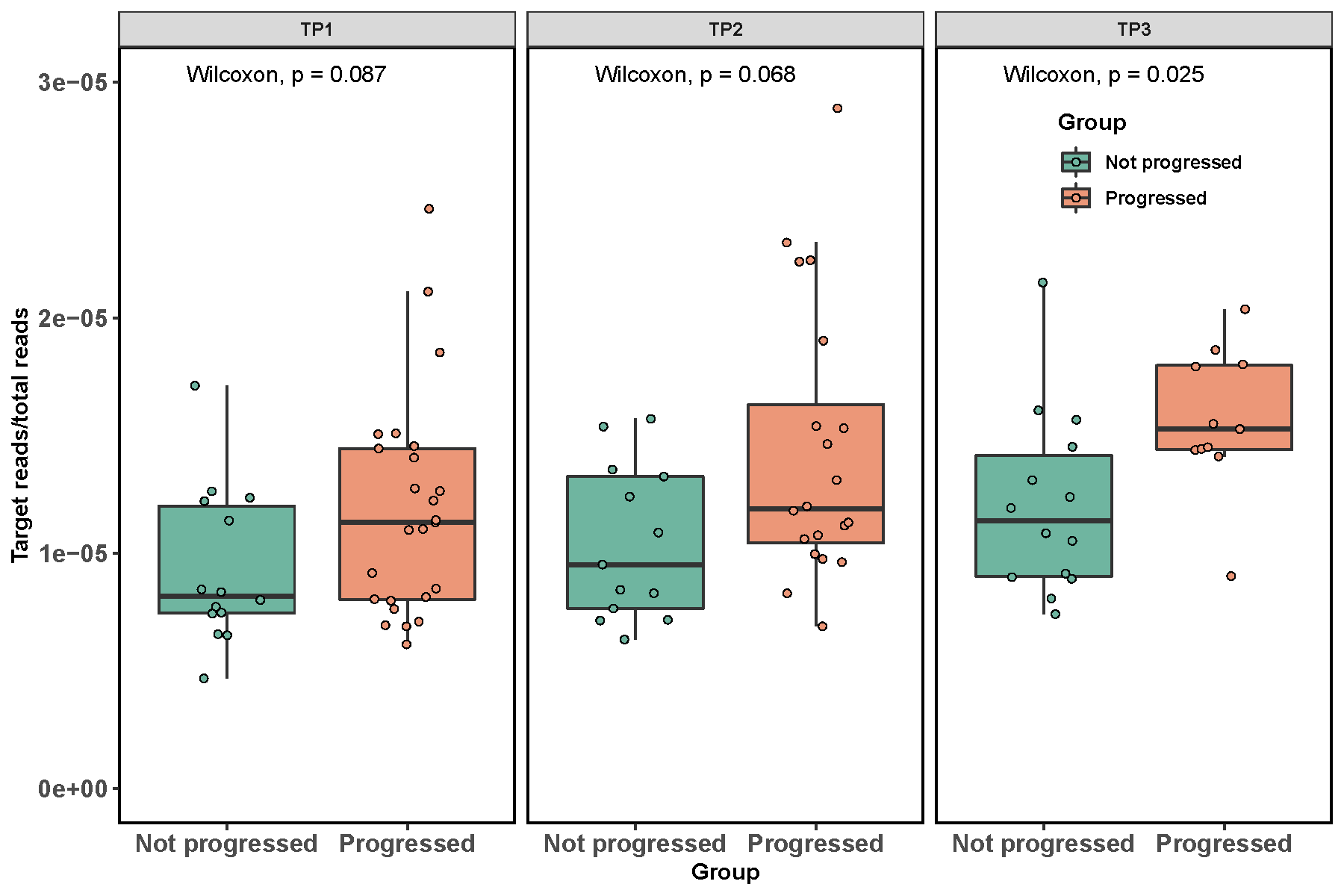
**Figure S1**. **Neomer feature pattern at different time points.** Boxplots showing the ratio of target reads and total read between the not yet progress patient group and progress patient group at A) TP1 B) TP2 and C) TP3. TP1: Week 4 of CRT/RT; TP2: 1 month after CRT/RT; TP3: 3 months after CRT/RT.


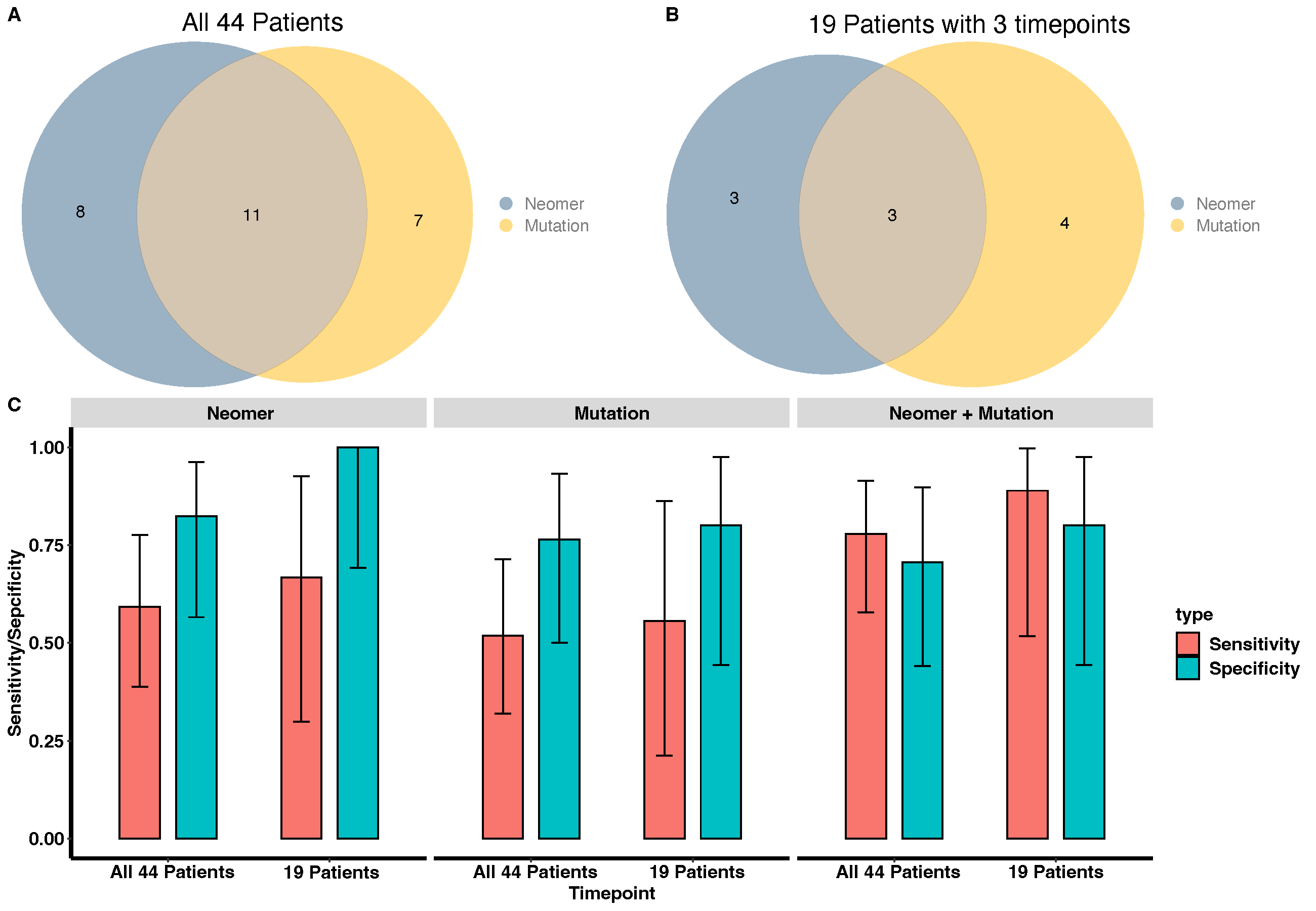


**Figure S2. Enhanced sensitivities in detecting high risk patients for disease progression using combined time points.** Venn diagrams of Neomer model prediction and ctDNA detection status for combined time points in A) all patients B) selected patients (all three time points available). C) Bar plots of sensitivities and specificities for detecting high progressive risk patients in the cohort. The error bars represent 95% confidence intervals.


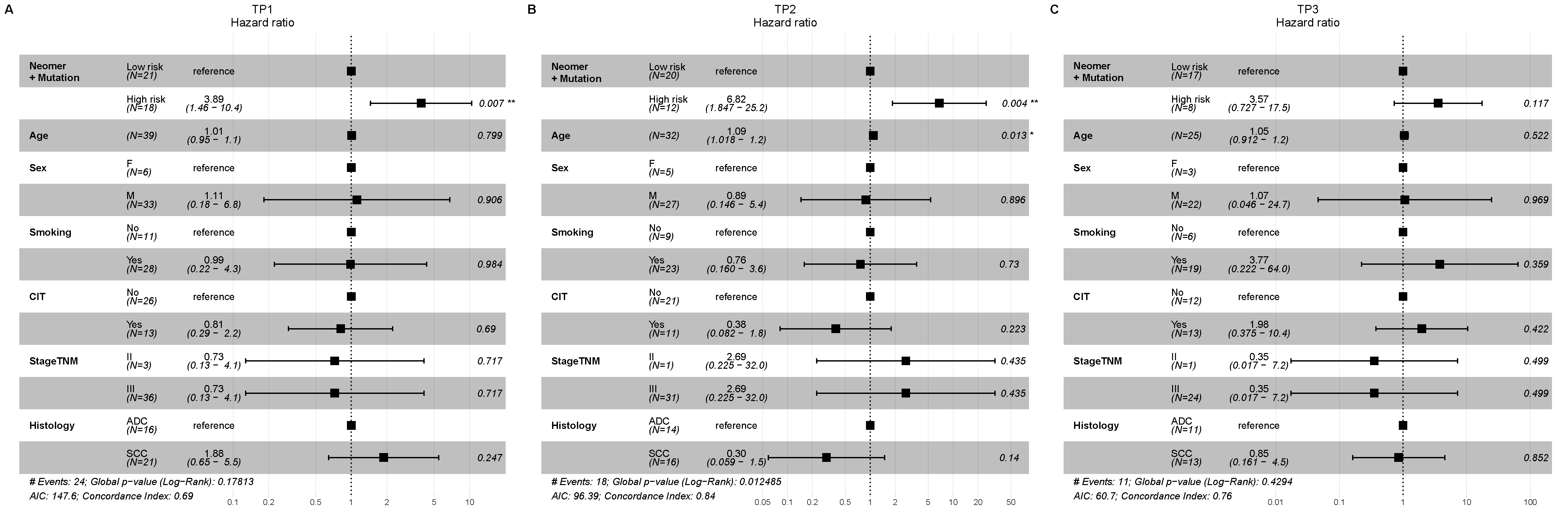


**Figure S3. Multivariate analysis for TP1, TP2 and TP3.** Forest plots for A) TP1; B) TP2; C) TP3. TP1: Week 4 of CRT/RT; TP2: 1 month after CRT/RT; TP3: 3 months after CRT/RT.


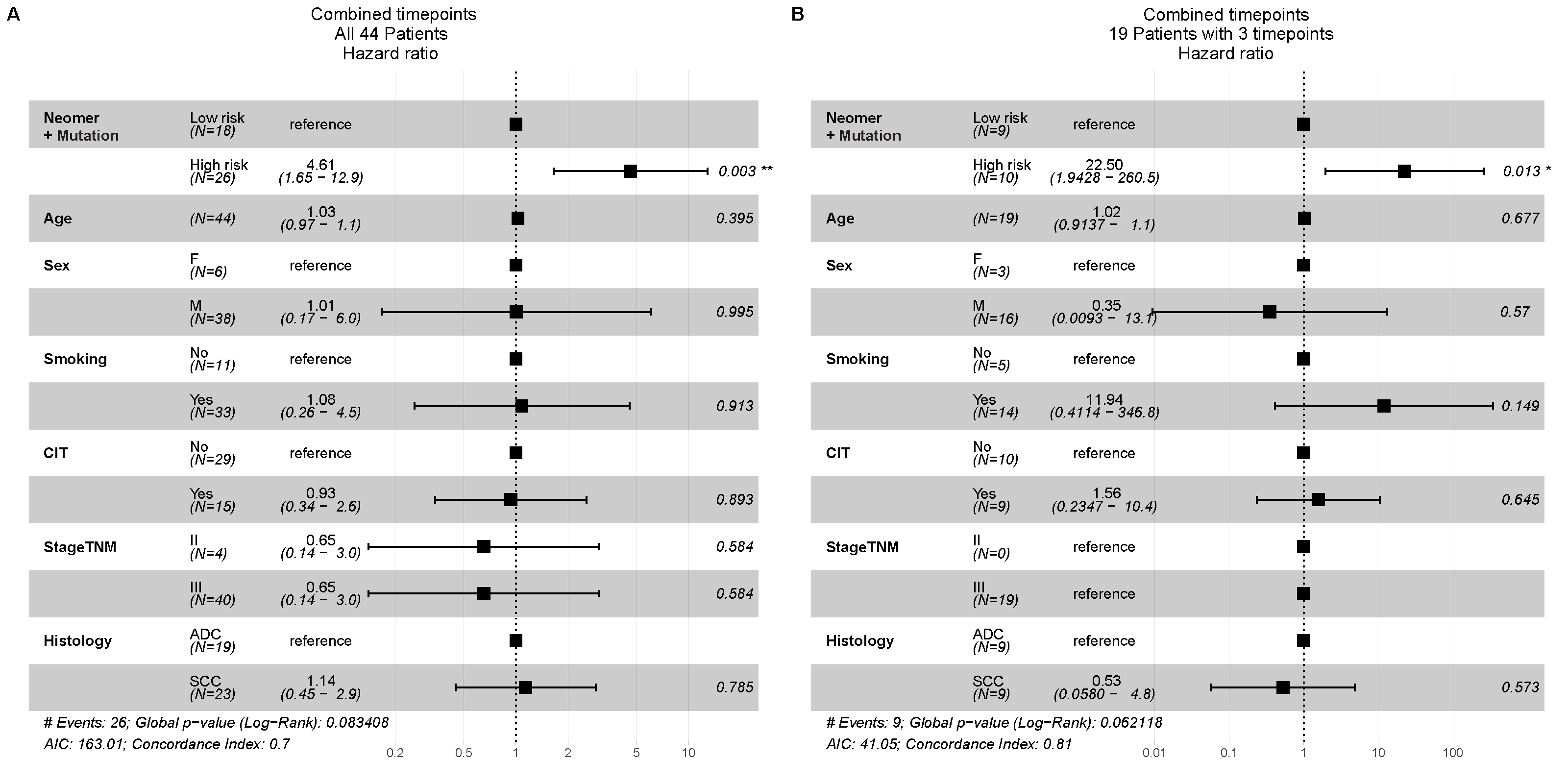


**Figure S4. Multivariate analysis for combined time points.** Forest plots for A) all 44 patients; B) selected 19 patients in the longitudinal sub cohort with 3 time points.

**
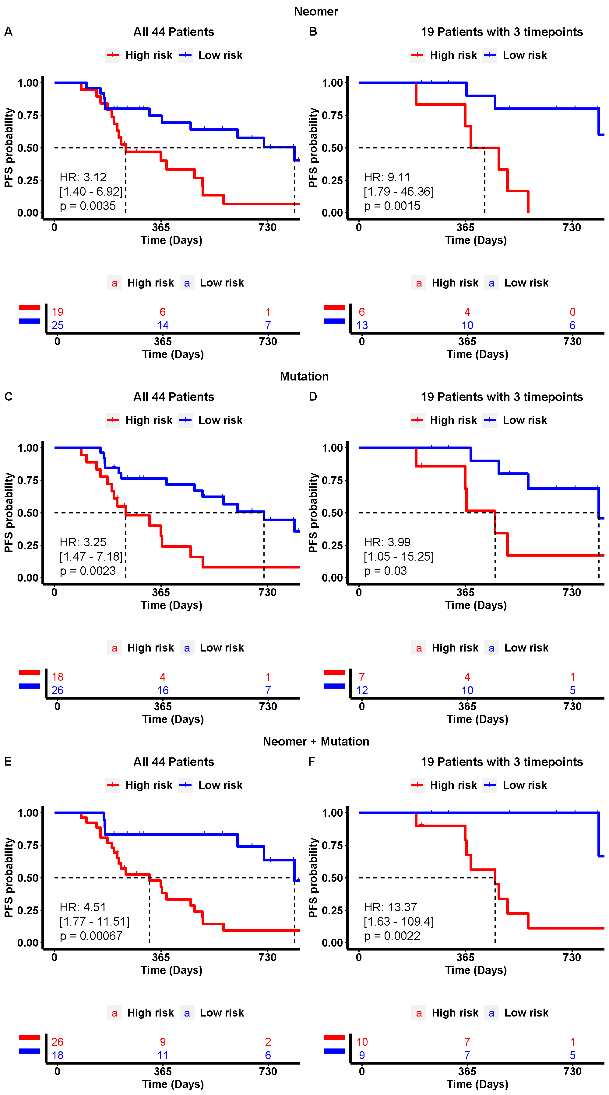
**

**Figure S5**. **Progress-free survival analysis using combined time points in localized inoperable NSCLC patients using Neomer and mutational methods.** Kaplan-Meier curves of progression-free survival stratified for combined time points (patient labelled as high risk if for any single high risk time point) in all patients by A) Neomer model predicted status for combined time points C) mutation based ctDNA status E) combination of Neomer model predicted and mutation based ctDNA status. Kaplan-Meier curves of progression-free survival stratified for combined time points (patient labelled as high risk if for any single high risk time point) in selected patient group (patients have all three time points) by A) Neomer model predicted status for combined time points C) mutation based ctDNA status E) combination of Neomer model predicted and mutation based ctDNA status.


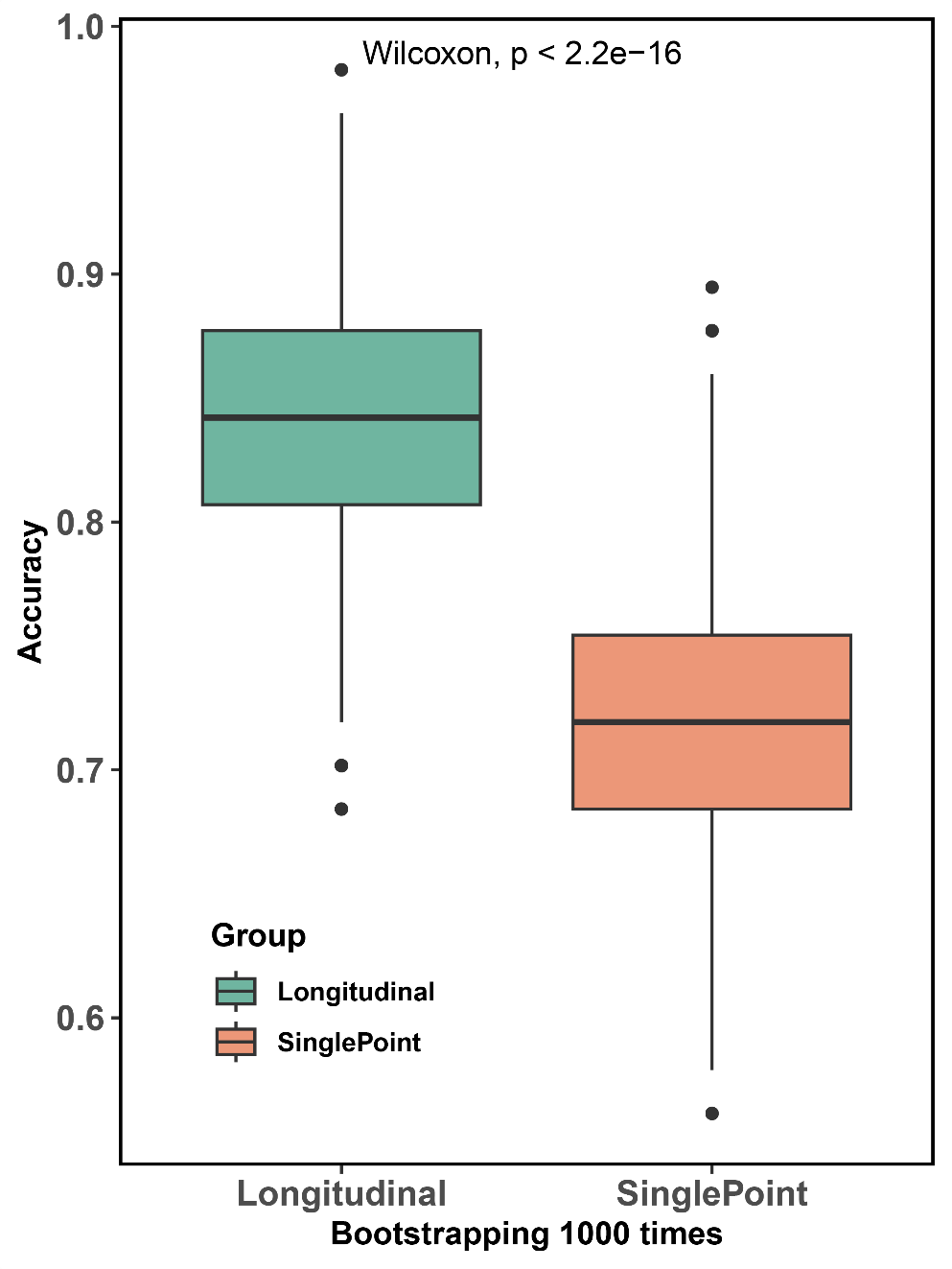


**Figure S6. Boxplot of overall accuracies of longitudinal data and single time point data.** The bootstrapping analysis (n = 1,000) were performed on the total 57 samples from 19 patient who were assessed at all three time points.
